# Appraisal, Work-Up and Diagnosis of Anterior Uveitis: A Practical Approach

**DOI:** 10.4103/0974-9233.58416

**Published:** 2009

**Authors:** Carl P. Herbort

**Affiliations:** Retinal and Inflammatory Eye Diseases, Centre for Ophthalmic Specialized Care and University of Lausanne, Lausanne, Switzerland

**Keywords:** Anterior uveitis, Uveitis work-up, Granulomatous uveitis, Non-granulomatous uveitis

## Abstract

This article presents a comprehensive approach of the diagnosis of anterior uveitis and appropriate investigational tests based on clinical signs.

Uveitis has classically been presented by uveitis specialists as an obscure and complicated field in ophthalmology that was supposed to be restricted to the happy few who had the knowledge, which in some countries was even prevented from being diffused. The effect was that ophthalmologists turned away from uveitis or were not correctly armed when they chose to take care of uveitis patients. The consequences of this situation often fell upon the patients. Since more than 15 years our group has been represented by the Society for Ophthalmo-Immunoinfectiology in Europe (SOIE), which has been working to alter this image of uveitis and ensure that the knowledge of the basics of uveitis reaches the practicing ophthalmologist. Our firm believe is that up to 70% of uveitis cases, especially anterior uveitis, can be taken care of by the practicing ophthalmologist following a structured approach in the appraisal of the uveitis case. Judging from the attendance obtained, the response to our approach in every country (where we organise courses) has been inversely proportional to the previous disinterest since we started publicizing it.

## INTRODUCTION

**Figure 1a and b:**
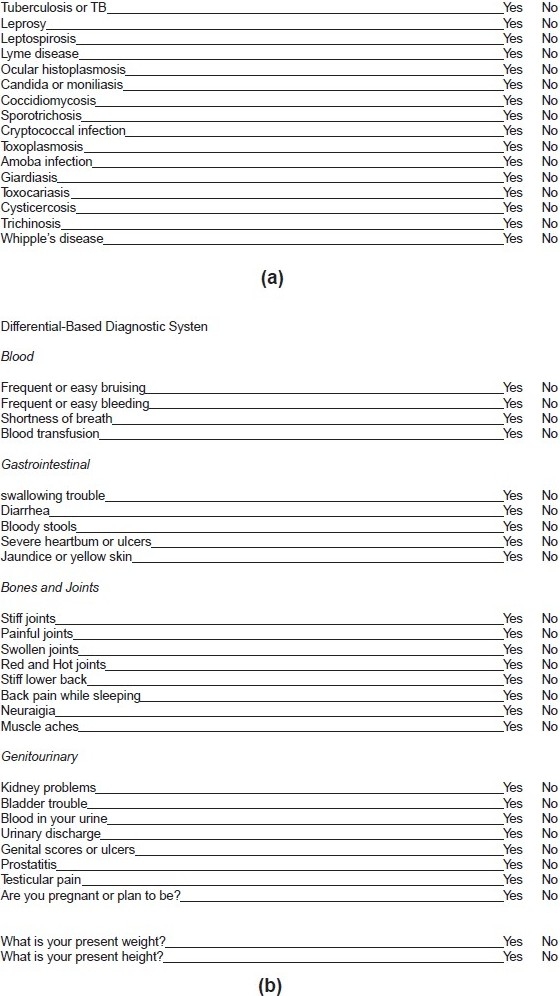
Example of the traditional lists that are featured in most uveitis books, which generate more confusion than help

The diagnostic yield in uveitis has significantly improved in the last 15 to 20 years, in part because a clear classification is available to the clinician but even more so because diagnostic tests have greatly improved lately. With a systematic approach, based on history and clinical examination and guided by laboratory tests and other investigational tests, a diagnosis can be expected in about 70% of cases. A compilatory approach, as exposed in most uveitis textbooks, has to be avoided [Figures 1a and b]. The endless lists of disease, exposure which the patient is supposed to tick on a check-list do not contribute to a diligent work-up of uveitis and are even counterproductive as this may push the clinician in the wrong direction.

It is much more productive to use a comprehensive approach that avoids a detailed anamnesis (history) at the beginning of the consultation but limits the initial anamnesis (history) to the immediate complaints of the patient followed immediately with a thorough clinical examination. Once the clinical signs have been established and have allowed to characterize the type of uveitis (granulomatous versus non-granulomatous) the anamnesis (history) can be resumed by asking specifically directed questions. The clinician should then try to classify the uveitis more precisely, refer to the local epidemiological data and come up with a working diagnosis, which he should always be prepared to change. At this point, targeted investigations and laboratory tests should be performed. The result is a diagnosis which has a higher degree of probability that can be followed by appropriate therapy. In case no diagnosis is suspected, nonspecific therapy may have to be started or therapy can be withheld. In a proportion of cases the evolution of the disease leads to the diagnosis [Flow-chart 1].

### Work-up of anterior uveitis [Flow chart 1]

#### Definition

The anatomical classification of uveitis into anterior, intermediate and posterior forms is very useful to conduct the work-up and eventually to reach a diagnosis, even though inflammation does not always respect these anatomical boundaries. Anterior uveitis is the term used for the group of inflammatory disorders for which the preponderant part of the inflammation is situated at the level of the pars plicata of the ciliary body, the retroiridal space, the iris and the anterior chamber.

#### Symptoms and signs of anterior uveitis [Flow chart 2 and 3]

The severity of symptoms in anterior uveitis ranges from no symptoms in chronic disease such as anterior uveitis related to juvenile idiopathic arthritis (JIA) to very severe symptoms in acute uveitis such as HLA-B27 related uveitis. Symptoms of acute anterior uveitis include photophobia, redness, pain, decreased vision and tearing in the absence of discharge.

The signs of anterior uveitis are listed in Table 1:

**Table 1:** Signs of anterior uveitis

**Figure 2:**
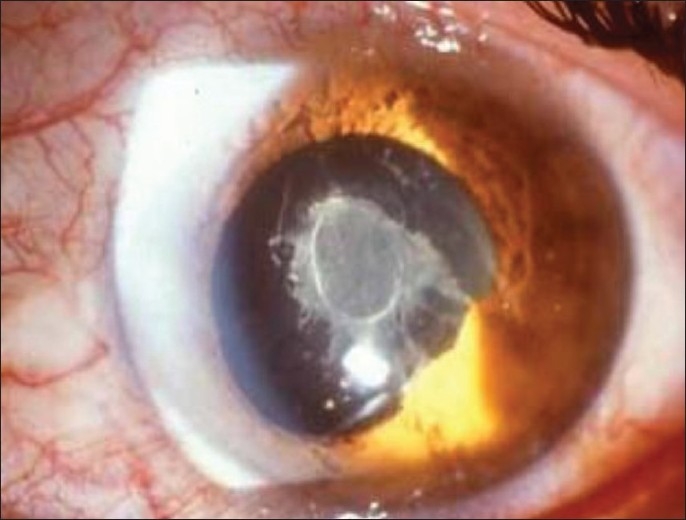
Fibrinous clot in anterior chamber in typical case of HLA-B27 related uveitis

Conjunctival injection
Keratic precipitates
Aqueous flare/fibrinous clots [Figure 2]
Posterior synechiae between the iris and the capsule of the lens [Figure 3]
Aqueous cells/hypopyon [Figure 4]
Iris rubeosis (usually reversible)
Iris nodules (Koeppe/Busacca) [Figures 5a and b]
Iris atrophy (herpes uveitis and Fuchs' uveitis)
Intraocular pressure changes (hypotony in severe acute anterior
non-granulomatous uveitis; hypertony in granulomatous uveitis)

1. The conjunctival injection in anterior uveitis can be diffuse or localized circumferentially at the limbus (perikeratic injection) or mixed (diffuse and perikeratic injection).
2. The morphology of keratic precipitates (KPs) is very useful to help distinguish non-granulomatous from granulomatous uveitis. Small diffuse KPs causing dusting of the endothelium are characteristic for non-granulomatous uveitis such as HLA-B27 related acute anterior uveitis. When KPs become larger than endothelial dust they can be individualised and correspond to granulomatous KPs. The morphology and distribution of granulomatous KPs is often useful in orienting towards a more specific diagnosis within granulomatous causes [Table 2]. Medium and large size KPs are called “mutton fat” KPs. There is a lot of confusion regarding the characterisation of KPs. In many textbooks only mutton-fat KPs are termed granulomatous and small granulomatous KPs as those found in Fuchs' uveitis are erroneously termed as non granulomatous. Therefore, Fuchs' uveitis is mistakenly classified as non-granulomatous in many textbooks. It is useful to distinguish between fresh and chronic (old) muton-fat KPs. Old mutton-fat KPs tend to be less white, pigmented and less dense in the center.

**Table 2:** Granulomatous keratic precipitates

Type of KP Small stellate, even distribution no gravitation	Clinical entity to suspect Fuchs' uveitis [Figure 6]
Small gravitational or inferior random distribution	CMV uveitis [Figure 7]
Small/medium/size, focal spherical distribution under stromal keratitis	h.simplex/zoster keratouveitis [Figure 8]
Medium/large gravitational distribution (mutton-fat)	sarcoidosis, tuberculosis, toxoplasmosis [Figure 9]
Very few (2–5) small/medium/large size, inferior peripheral (iridocorneal angle)	Posner-Schlossmann syndrome
3. Anterior chamber flare is caused by exudation of proteins into the normally clear aqueous humor from iris vessels or across the ciliary body epithelium following the breakdown of the blood-aqueous barrier. The intensity of flare is measured in a standard fashion following the grading system proposed by the Proctor Group in San Francisco in 1959 [Table 3].^1^ It is, however, only qualitative. A beam 1 mm wide and 3 mm long with the highest light intensity and × 16 magnification on the BQ Haag-Streit slitlamp is used. When the concentration of proteins in the aqueous is very high, they agglomerate and form fibrinous clots, a finding more common in acute non-granulomatous uveitis [Figure 2].

**Table 3:** Slit-lamp grading of aqueous flare (1 mm × 3 mm beam)

No flare	0
Faint, just detectable	+
Moderate, iris details clear	+ +
Marked, iris details hazy	+ + +
Intense, fibrin	+ + + +
4. Depending on the amount and composition of aqueous inflammatory proteins adherences between the iris and anterior capsule of the cristalline lens can form (posterior synechiae) [Figures 3 and 4].

**Figure 3:**
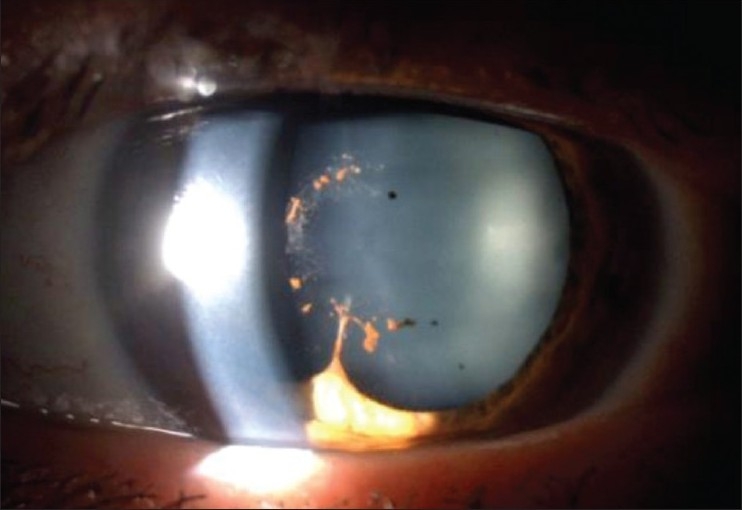
Posterior irido-lenticular synechiae-the ring of pigment deposits on the cristalline lens show where the iris was attached (synechiae). One remaining synechia at six o'clock on the verge of detaching itself following the administration of massive dilating drops
  Since a few years it has been possible to measure flare in a quantitative and objective fashion, using laser flare photometry (LFP) [Figure 10]. This new technology makes flare the only quantitative parameter to measure intraocular inflammation. So far, cells were judged more accurately to measure inflammatory activity in uveitis. At best this measurement is however only semi-quantitative. LFP was shown to be more sensitive than slit-lamp assessment of cells to measure the evolution of inflammatory activity, making flare the new gold standard to assess intraocular inflammation. LFP allows detecting subclinical flare intensities and changes, which can be predictive of clinical recurrence. It allows detecting resistance of inflammation to treatment and closer follow-up of therapy, often leading to corticosteroid sparing in the treatment. When available, laser flare photometry certainly allows improved management of uveitis.^2^
5. Aqueous cells used to be the reference parameter for inflammatory activity because their evaluation was quantifiable by slit-lamp examination. Nowadays this is no more true when LFP is available. LFP is the quantifiable gold standard to measure inflammatory activity even in chronic inflammation with chronic breakdown of the hemato-ocular barriers, as it has been shown that even when there are no cells; LFP can detect active inflammation that responds to therapy. Grading of cells in the anterior chamber has been standardized by Hogan *et al*. at the Proctor Foundation in 1959^1^Table 4.
  It is important to make the difference between pigment clumps and inflammatory cells and examine the anterior chamber prior to mydriasis as cells and especially pigment dispersion can sometimes be seen after pupillary dilatation. When the quantity of cells is very dense they sediment and cause a hypopyon, a sign more often seen in HLA-B27 related uveitis, Behçet's uveitis and uveitis related to juvenile idiopathic arthritis (JIA) [Figure 4].

**Figure 4:**
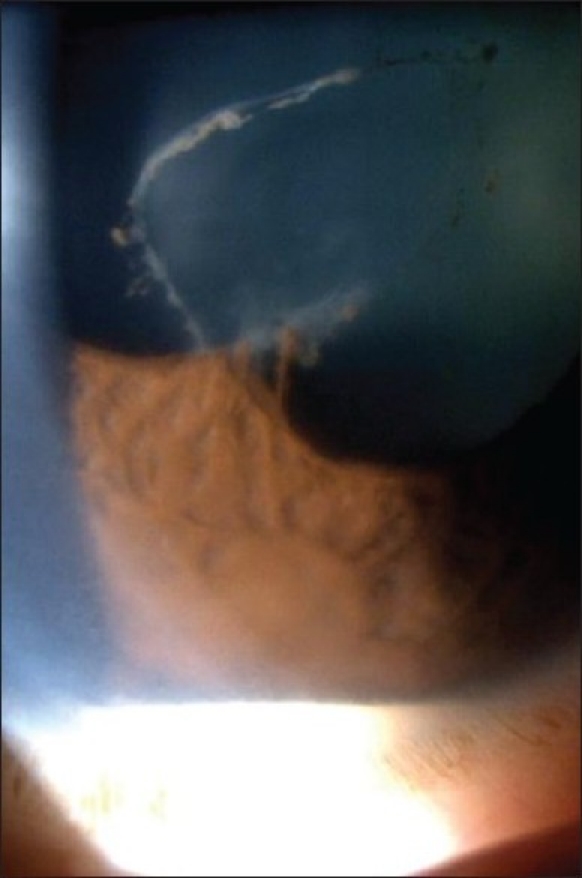
Hypopyon: Sedimented white cells form a level at the bottom of the anterior chamber, associated with a ring of fibrine on the surface of the cristalline lens (top) indicating broken synechiae. One remaining synechia on the meridian of 7 o'clock
6. In severe and longstanding uveitis iris rubeosis can develop. It is in fact most often a pseudo-rubeosis that is reversible after introduction of anti-inflammatory treatment. Even when a real rubeosis has developed, it is usually situated at the pupillary border of the iris and is much less agressive and proliferative than ischemic rubeosis iridis. In Fuchs' uveitis with extensive iris atrophy iridal vessels can be seen and correspond to a pseudo-rubeosis.
7. Iris nodules [Figures 5a and b]

**Figure 5:**
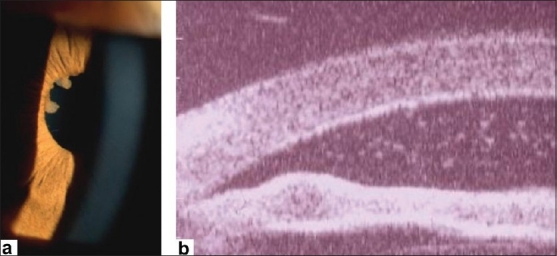
(a) Koeppe nodules: Two Koeppe nodules at the border of the iris associted with synechiae; (b) Busacca Nodules: UBM picture showing nodule within the stroma in a patient with tuberculous uveitis
  Two types of iris nodules can develop in granulomatous uveitis. When situated at the pupillary margin (and on the surface of the iris) they are called Koeppe nodules, have a fluffy appearance and a size going from very small barely visible excresences to frank nodules. When situated in the body of the iris stroma, iris nodules are called Bussaca nodules.
8. Sectorial or widespread iris atrophy is a sign rather specific for herpes simplex or herpes zoster uveitis and is a useful diagnostic help. Diffuse atrophy is very often seen in Fuchs' uveitis.
9. Intraocular pressure changes due to uveitis can present either as hypotension or hypertension. Hypotony is usually measured in severe uveitis involving the ciliary body such as acute anterior non-granulomatous HLA-B-27 related uveitis. Hypertony is usually associated with granulomatous uveitis, especially herpes simplex or herpes zoster uveitis but also sarcoidosis or spill-over anterior uveitis associated with toxoplasmic retinochoroiditis. In recent years, CMV anterior uveitis, a newly recognized usually unilateral entity very often causing hypertony has been described.^3^

**Table 4:** Slit-lamp grading of aqueous cells (1 mm × 3–4 mm beam)

No cells	0
1–5 cells	±
6–10 cells	±
11–20 cells	+ +
21–50 cells	+ + +
> 50 cells	+ + + +

### Work-up of anterior uveitis [Flow chart 2–4]

The anatomical diagnosis of anterior uveitis has to be first verified by excluding spill-over inflammation associated with uveitis of the posterior segment (intermediate or posterior uveitis). To exclude posterior involvement pupil dilatation is mandatory in all cases. Secondly, the type of clinical presentation has to be characterized as non-granulomatous or granulomatous in order to correctly orient work-up and differential diagnosis.

Non-granulomatous uveitis is characterized mainly by the type of keratic precipitates that presents as fine KPs producing endothelial dusting. In severe cases fibrinous clotting or hypopyon can occur depending on whether protein influx or cellular infiltration is predominant. In case of severe inflammation it is also common to find posterior synechiae and pressure tends to be more often decreased than increased [Table 5].

**Table 5:** Common causes of non-granulomatous anterior inflammation in anterior uveitis or panuveitis

HLA-B27 related uveitis
Behçet's uveitis
Juvenile rhumatoid arthritis related uveitis
Uveitis associated with scleritis
Uveitis associated with streptococcal infection

Granulomatous uveitis is characterized by KPs that are larger than the dusty KPs of non-granulomatous uveitis. They are better individualized but their size varies depending on the inflammatory process. The medium and large size granulomatous KPs are called mutton-fat KPs [Figure 9]. Other characteristic features of granulomatous uveitis are Koeppe and Bussaca nodules [Figures 5a and b]. Synechiae are common in more pronounced inflammation. Pressure changes when present are usually characterized by increased intraocular pressure.

Although this distinction is a very useful working classification, the subdivision is not an absolute one. A granulomatous uveitis may initially present as nongranulomatous when dusty KPs are very thick, i.e, before considering its granulomatous aspect. Conversely, in rare cases a non-granulomatous uveitis can take an aspect that might be qualified as granulomatous. In most cases the distinction is however rather well delineated [Table 6].

**Table 6:** Common causes of granulomatous anterior inflammation

In anterior uveitis
Fuchs' uveitis
Herpetic (herpes simplex, varicella-zoster, Epstein-Barr) uveitis
CMV anterior uveitis
Posner-Schlossmann syndrome
Sarcoidosis
Tuberculosis
Syphilis
In panuveitis
Sarcoidosis
Vogt-Koyanagi-Harada syndrome, sympathetic ophthalmia
Tuberculosis
Syphilis
Toxoplasmic retinochoroiditis
Necrotizing herpetic retinopathies (acute retinal necrosis)
Post-surgical inflammatiom (propionibacterium acnes)

### Work-up of non-granulomatous anterior uveitis

In case of simple, fibrinous or hypopyon nongranulomatous uveitis, the only first-line work-up test we presently perform is the detection of the HLA-B27 antigen. HLA-B27 testing is performed even if the inflammation is only moderate. If the test is positive it avoids further unnecessary testing during a subsequent episode and it is reassuring for the patient and the doctor to know the specific diagnosis, especially when it is a benign disease. In case of a positive result no further investigation is performed at the ophthalmological level. It is, however, recommended to take an oriented history that allows, with the help of the internist or rheumatologist to sub classify the condition as ankylosing spondylarhritis, Reiter's syndrome, Crohn's disease, ulcerative colitis or simply into HLA-B27 uveitis without systemic associated disease when necessary. About 50–55% of acute anterior non-granulomatous uveitis is HLA-B27 positive in Europe, but this varies from one geographical area to another, being for instance quite low in Japan. In the remaining 45–50% of cases a specific diagnosis is more difficult to establish.^4^

No further investigation is performed if the episode of HLA-B27 negative non-granulomatous uveitis is of limited severity and/or responds readily to topical corticosteroid therapy.

In case of an anterior uveitis with hypopyon, signs and symptoms found in Behçet's syndrome should be investigated, in particular oral and/or genital ulcerations, cutaneous signs such as erythema nodosum and pustules, arthralgies, thrombophlebitis or central nervous system involvement. If Behçet's uveitis is suspected we find it useful to look for the HLA-B51 antigen which, when present, represents an aditional argument for the diagnosis of Behçet's uveitis, especially in the milder forms of Behçet's uveitis seen in the Caucasian European population. Isolated anterior Behçet's uveitis can occur but posterior involvement should be searched for by funduscopy and is best investigated by performing a fluorescein angiography looking for retinal vasculitis.

In case of non-granulomatous uveitis in children (with or without band keratopathy) history should be directed towards juvenile idiopathic arthritis (JIA). Inflammatory symptoms can be completely absent, contrasting with the severe signs of uveitis such as hypopyon and extensive synechiae that can characterise JIA related uveitis. Uveitis is usually associated with the pauciarticular form of JIA and testing should include anti-nuclear antibodies (ANA) that are present in up to 70% of JRA patients with uveitis. In elderly children it is also useful to test for the presence of HLA-B27 antigen.

A bilateral nongranulomatous uveitis in children, but also in adults, should prompt to search or exclude tubulointerstitial nephritis and uveitis syndrome (TINU), an often neglected diagnosis. Renal function should be tested, starting with the dosage of creatininemia requiring sometimes renal biopsy and urinalysis should be performed looking for glucosuria and dosage of beta-2-microglobulin which is found to be elevated in TINU.^5^

In children, pars planitis can initially present with a pronounced anterior participation and can be mistaken for an anterior uveitis if the posterior segment is not carefully analyzed.

In case of non-responding HLA-B27 negative anterior uveitis or in case of recurrence we pursue the work-up in the same fashion as for a granulomatous uveitis.

### Work-up of granulomatous uveitis

**Figure 6:**
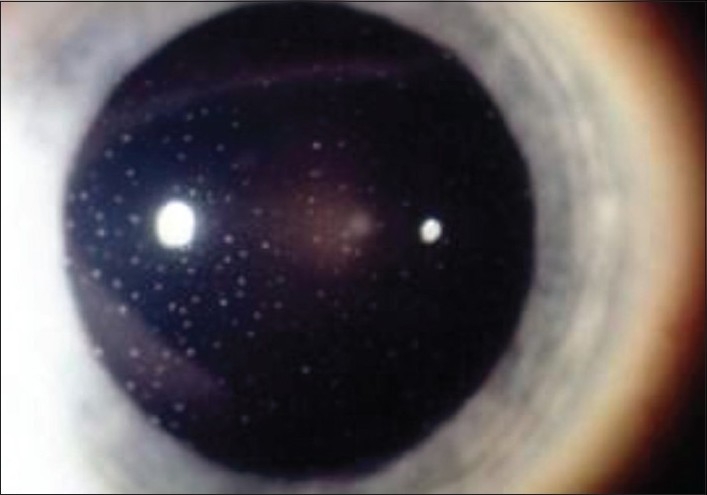
Fuchs uveitis: Small stellate non-gravitationally distributed keratic precipitates (KPs) typical of Fuchs' uveitis

Before starting the work-up of granulomatous uveitis, it is important to exclude Fuchs' uveitis as this condition, when sufficiently typical, does not need any work-up. Further, corticosteroid treatment should be withheld in Fuchs' uveitis to avoid the side-effects of a treatment that usually has no impact on the inflammatory process. Characteristic findings of Fuchs' uveitis include fine stellate granulomatous keratic precipitates which usually do not accumulate inferiorly by gravitation but are more uniformly distributed over the whole surface of the endothelium, [Figure 6] fine Koeppe nodules at the pupillary edge of the iris, prominent vessels in the irido-corneal angle seen by gonioscopy and absence of posterior synechiae. Heterochromia is only present in fair-colored irises not dark irises. Therefore, the term heterochromic should be deleted for this entity and it should simply be called Fuchs' uveitis. It should be kept in mind that involvement can be bilateral. Laser flare photometry shows a very moderate breakdown of blood-aqueous barrier of 10.2 ± 3.5 ph/ms (normal 3.5 – 4.0 ph/ms) that remains relatively stable over time and usually does not respond significantly to anti-inflammatory treatment.

**Figure 7:**
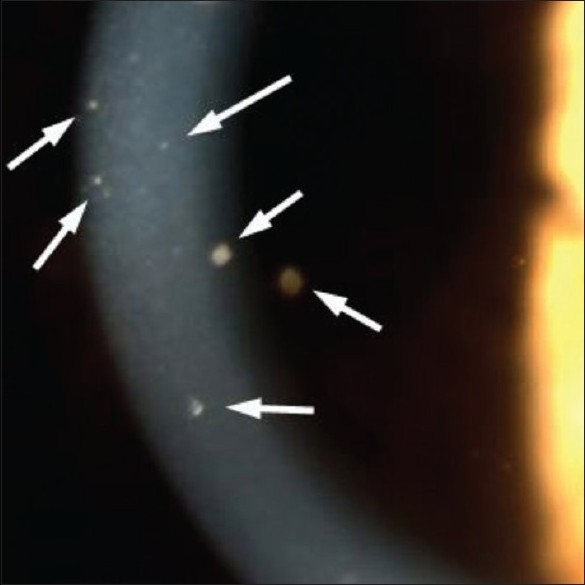
KPs in CMV anterior uveitis: Scarce unilateral KPs of different sizes (small to medium) in a case of cytomegalovirus anterior uveitis

**Figure 8:**
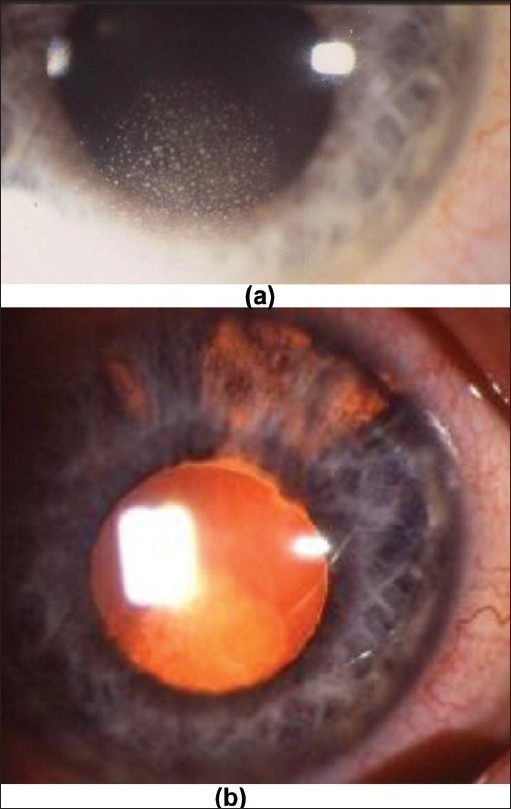
(a) Herpes KPs: Usually herpes/zoster KPs arranged in a round disciform pattern; small to medium sized; (b) Herpes KPs: Usually arranged in a round disciform pattern, very well visible against retroillumination (Note also sectorial iris atrophy at the top of the picture at the level of the superior iris)

**Figure 9:**
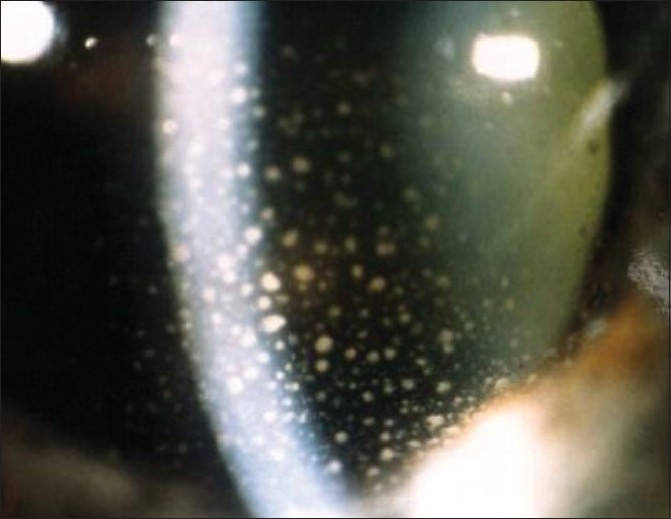
Mutton-fat KPs: This type of gravitational large KPs can be seen in tuberculosis and sarcoidosis. In toxoplasmosis the anterior inflammation sometimes associated with the retinitis is also made of large mutton-fat KPs, although the KPs are usually less numerous

**Figure 10:**
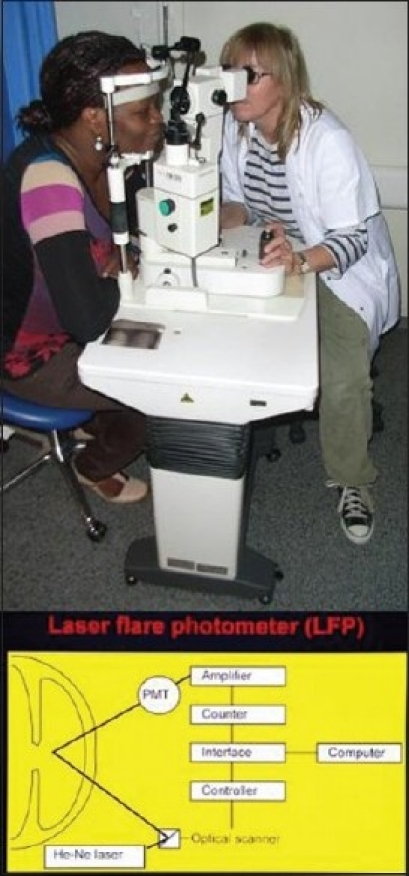
Laser flare photometry: This machine measures the content of proteins in the anterior chamber and hence the level of inflammation in an objective and quantitative fashion. The instrument shines a laser into the anterior chamber and the electronic detector is counting the number of photons that are back-scattered. Back-scattered photons are proportional to the amount of particles (proteins) and hence proportional to the intraocular inflammation

**Flow-chart 1:**
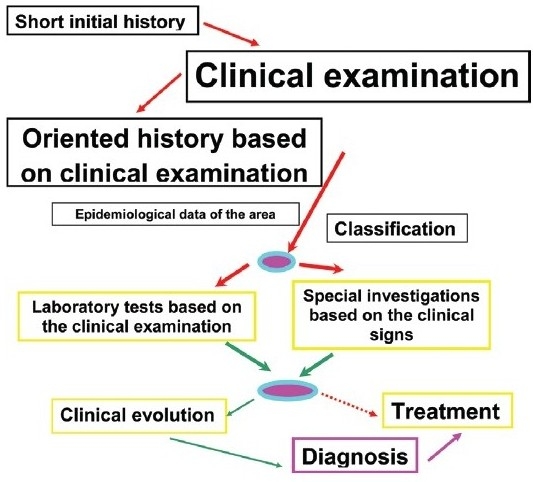
General principles in the appraisal of a uveitis case: proceed with guidance of the clinical signs

**Flow-chart 2:**
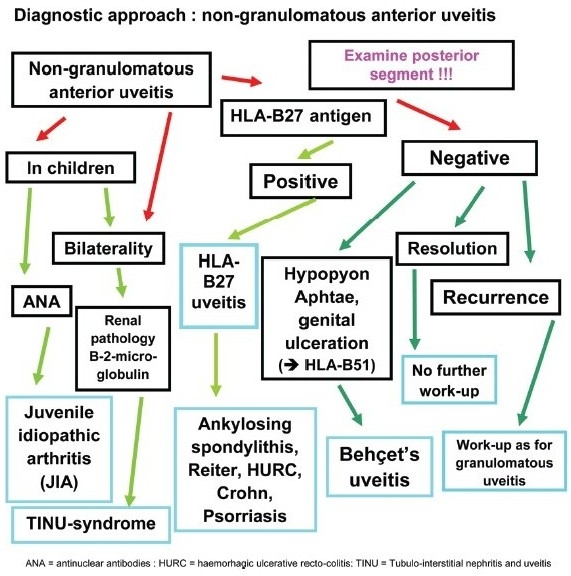
Diagnostic steps in non-granulomatous uveitis

**Flow-chart 3:**
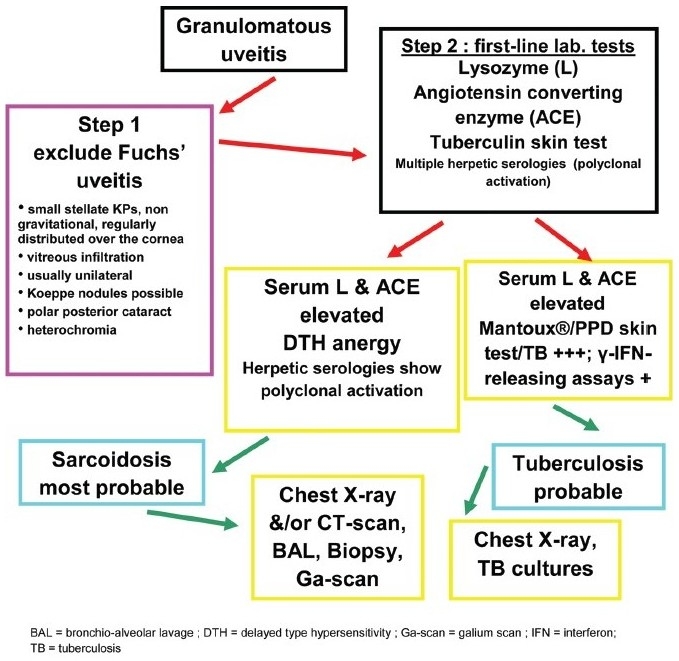
Diagnostic steps in granulomatous uveitis

**Flow-chart 4:**
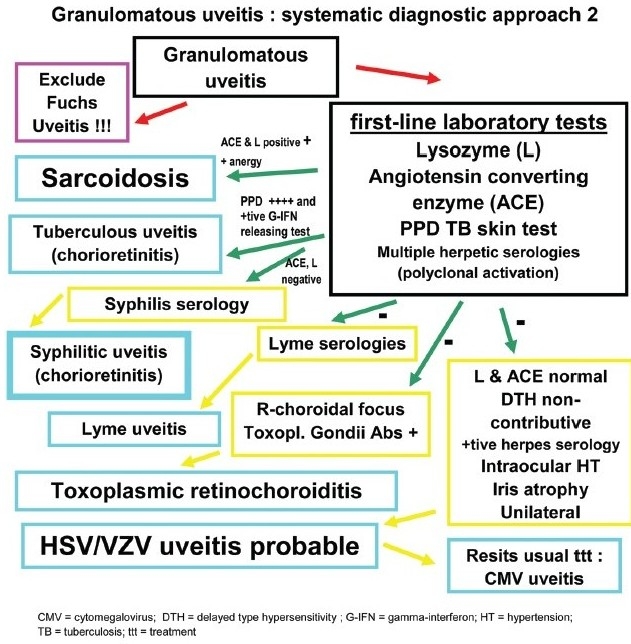
Differential diagnosis of granulomatous uveitis

Spill-over anterior granulomatous uveitis can occur in very inflammatory toxoplasmic retinochoroiditis which has to be excluded by performing a detailed examination of the posterior segment with funduscopy.

The first-line laboratory tests performed to investigate granulomatous uveitis are serum angiotensine converting enzyme (ACE) and lysozyme, products indicating the presence of granulomatous lesions. ACE can be normally elevated in children and serum lysozyme levels tend to be progressively more elevated in elderly persons. It is therefore important to perform both tests.

The second step is to differentiate between sarcoid, tuberculous or other granulomatous causes. In order to differentiate between tuberculosis and sarcoidosis, multiple skin tests measuring delayed type hypersensitivity reaction to several antigens to which the adult patient should be normally reacting (MultiMérieux® containing tuberculin, streptococcus, diphteria, tetanus, tricophyton and candida antigens) are performed to search for anergy, a strong argument for sarcoidosis. These tests are, however, rarely performed nowadays as the Merieux® multi-antigen applicator is difficult to find. In a patient vaccinated for tuberculosis or known to have been exposed to a tuberculous infection a PPD skin test which has become negative has the same diagnostic value. We find it also useful to look for polyclonal antibody activation that is present in up to 85% of the patients with sarcoidosis^6^,^7^ and was also found in patients with ocular sarcoidosis.^8^ For this purpose, serologies to four herpes viruses to which most of the adult population has been exposed (herpes simplex, herpes zoster, cytomegalovirus and Epstein-Barr) are performed. ELISA serology detects exposure to these viruses and complement fixation serology is done to establish whether the antibody titers are elevated. An isolated elevated titer to only one virus might be indicative of a viral etiology.

Polyclonal activation, however, is an additional element for sarcoidosis. This non-specific antibody elevation is the cause of some of the false-positive diagnosis of presumed infectious uveitis, relying only on a serology such as Lyme borreliosis. A positive serology is not a confirmation of ocular Lyme disease. We followed five cases with uveitis and a positive Lyme serology that had negative anterior chamber antibody ratios (Goldmann-Witmer coefficient) and for whom the diagnosis was finally sarcoidosis. Patients with a compatible clinical picture and positive ACE and lysozyme tests in presence of cutaneous anergy have a probability of over 95% to have ocular sarcoidosis.^9^

On the other hand, when the PPD tuberculin skin test is hyperpositive, this should raise the suspicion of a tuberculous granlomatous uveitis. The next test to be performed is one of the gamma-interferon releasing assays which test blood lymphocytes of patients in order to detect lymphocytes reacting *in vitro* when put in the presence of specific proteins from *Mycobacterium tuberculosis*. When the patient's lymphocytes release gamma-interferon, it means that the patient has been exposed to the bacteria and tuberculosis should be actively researched.^10^

Syphilis serology is performed either routinely or in case of a positive history. In case of undefined diagnosis, serology for Lyme borreliosis is performed with the known limitations of the value of a positive serology. Toxoplasmic retinochoroiditis can sometimes present as a granulomatous (hypertensive) anterior spill-over uveitis. The presence of a retinal focus orients clearly into this direction. In order to make the diagnosis possible toxoplasmic serology should be performed to show the presence of IgG antibodies indicating that the patient has been in contact once in his life with *Toxoplasma gondii*.

In case of negative ACE/lysozyme test and a non-contributary skin test a herpetic uveitis should be suspected. Clinical signs that are very suggestive of herpes simplex/zoster uveitis are ocular hypertension and iris atrophy (found both in herpes simplex and varicella-zoster uveitis). Laboratory confirmation of herpes simplex/zoster anterior uveitis can be obtained by the detection of intraocular production of antibodies in the aqueous humor (Goldmann-Witmer coefficient). Aqueous paracenthesis is, however, not performed routinely in these cases but reserved for sight-threatening diseases such as necrotic herpetic retinopathies (NHR) that include acute retinal necrosis. It is also performed in uveitis suspected to be herpetic but that does not respond to classical combined systemic antiviral and topical steroidal therapy to detect CMV DNA in the aqueous.

A condition that can be associated with anterior granulomatous uveitis is multiple sclerosis (MS). In most cases, posterior segment findings such as periphlebitis and vitritis are usually present. In patients with a history compatible with MS, investigations should be directed towards MS beginning with a cerebral MRI.^11^
